# Alpha-Ketoglutarate Promotes Goblet Cell Differentiation and Alters Urea Cycle Metabolites in DSS-Induced Colitis Mice

**DOI:** 10.3390/nu14061148

**Published:** 2022-03-09

**Authors:** Alejandro Bravo Iniguez, Qiyu Tian, Min Du, Mei-Jun Zhu

**Affiliations:** 1 School of Food Science, Washington State University, Pullman, WA 99164, USA; a.bravoiniguez@wsu.edu (A.B.I.); qiyu.tian@wsu.edu (Q.T.); 2 Department of Animal Science, Washington State University, Pullman, WA 99164, USA; min.du@wsu.edu

**Keywords:** alpha-ketoglutarate, colitis, goblet cells, metabolome, mucin

## Abstract

The metabolite, alpha-ketoglutarate (aKG), shows promise as an approach for ameliorating colitis, but much remains unknown about the full extent of its effects on the metabolome and mucosal barrier. To further elucidate this matter, C57BL/6 male mice received drinking water with or without 1% aKG for three weeks, then were subjected to 2.5% dextran sulfate sodium (DSS) induction for 7 days followed by 7 days of recovery. Cecal content and intestinal tissue samples were analyzed for changes in metabolite profile and signaling pathways. Gas chromatography-mass spectrometry (GC-MS) metabolomics revealed a separation between the metabolome of mice treated with or without aKG; putrescine and glycine were significantly increased; and ornithine and amide products, oleamide and urea were significantly decreased. Based on a pathway analysis, aKG treatment induced metabolite changes and enriched glutathione metabolism and the urea cycle. Additionally, signaling pathways committing epithelial cells to the secretory lineage were elevated in aKG-treated mice. Consistently, aKG supplementation increased goblet cells staining, mRNA expression of mucin 2, and, trefoil factor 3 and Krüppel-like factor 4, markers of goblet cell differentiation. These data suggest the ameliorating the effects of aKG against chemically induced colitis involves a reduction in harmful metabolites and the promotion of goblet cell differentiation, resulting in a more-fortified mucus layer.

## 1. Introduction

Individuals suffering from inflammatory bowel disease (IBD) are at an increased risk of developing colorectal cancer, which is one of the leading causes of cancer-related death in the United States [1,2]. Normally, the intestinal epithelium maintains itself through a balance of proliferation, differentiation, and apoptosis, which is disrupted in IBD patients [3]. A combination of environmental and genetic factors contribute to the pathogenesis of IBD [4]. For example, the consumption of a high-fat diet aggravates colitis in mice through the promotion of oxidative stress and depletion of both goblet cells and mucin secretion [5,6]. In both children and adults, the prevalence of IBD has risen over the past decade making it a major concern [7]. Novel strategies are needed to reduce IBD incidence.

Dietary bioactive compounds suppress intestinal inflammation or dysfunction, showing preventive effects on IBD [8,9]. We previously demonstrated that dietary alpha-ketoglutarate (aKG), an intermediate of the tricarboxylic acid (TCA) cycle, ameliorates the severity of dextran sulfate sodium (DSS)-induced colitis, reducing intestinal damage by downregulating inflammatory pathways, promoting oxidative phosphorylation, and strengthening barrier function [10].

Proper cell differentiation is essential for a functional epithelial barrier [11], and epithelial differentiation is associated with a metabolic shift towards oxidative phosphorylation [12]. Dysregulated mitochondrial function hampers intestinal differentiation and, ultimately, barrier function [13,14]. In IBD, the signaling pathways that govern differentiation are altered [15]. Notch signaling promotes proliferation, and its ligands, including Jag1 and DLL1, are elevated in experimentally induced colitis [16]. Interestingly, while Notch signaling is found elevated in inflamed colonic tissue, it plays a vital role in ensuring proper epithelial regeneration, with its inhibition aggravating symptoms [17]. MATH1 commits intestinal epithelial cells to a secretory lineage, and transcription factors, such as Krüppel-like factor 4 (KLF4), are required for goblet cell differentiation [18]. Both MATH1 and KLF4 are negatively regulated by Notch signaling [19], and thus Notch often improves regeneration at the expense of goblet cell differentiation.

The differentiation of intestinal epithelial cells is also impacted by metabolites produced by the gut microbiome [20]. Different compounds present in the metabolome, such as short-chain fatty acids, vitamins, bile acids, and TCA cycle metabolites, promote either the proliferation or differentiation of epithelial cells [20]. Butyrate, produced by commensal gut bacteria fermentation, promotes the differentiation and expression of DNA mismatch repair genes [21]. Kynurenine, derived from tryptophan metabolism, suppresses Notch and promotes the differentiation of goblet cells in vitro [22]. Aspartate and fumarate are involved in a shunt connecting the TCA and urea cycles [23]. The urea cycle metabolites of major interest include polyamines, which display both beneficial and harmful effects on gut health. While polyamine levels are found to be elevated in colorectal tumors, they are also vital mediators of epithelial repair [24,25]. To the best of our knowledge, the effects of dietary aKG on the intestinal metabolome have not been widely explored. This study profiled the metabolite changes in DSS-treatment mice supplemented with or without aKG and further examined the impacts of aKG on pathways regulating epithelial differentiation.

## 2. Materials and Methods

### 2.1. Animals and Experimental Design

Male C57BL/6J mice at eight weeks of age were randomly assigned to control (CON) and aKG groups, receiving either 0% or 1.0% aKG in drinking water, respectively, during the entire dietary trial. On week 3 of aKG supplementation, mice were subjected to DSS treatment (7-day 2.5% DSS water induction followed by 7-day tap water) [10]. All animals survived the entirety of the study, and none had to be removed. Mice were sacrificed for tissue collection following recovery. Cecal content was collected at sacrifice and stored at −80 °C until needed. All animal procedures were approved by the Washington State University Institutional Animal Use and Care Committee (IAUCC, Pullman, DC, USA).

### 2.2. Tissue Collecting and Processing

Following anesthesia with CO_2_ and cervical dislocation, the intestine was dissected, and its contents were collected as previously described [10]. Samples were rinsed in phosphate-based saline (PBS) and stored at −80 °C except for a 5 mm segment of the colon, which was fixed in 4% paraformaldehyde. 

### 2.3. Histological Examination of Goblet Cells

Paraffin-embedded colonic tissues were cross-sectioned to 5 µm thickness, deparaffinized, rehydrated, and stained with Alcian Blue (pH 2.5) as previously described [26]. The staining of Alcian blue was quantified using Image J 1.30v software (split color channels) (National Institute of Health, Bethesda, MD, USA, https://imagej.nih.gov/ij/, accessed on 6 June 2021) for ratio of goblet cells/overall area [26].

### 2.4. RNA Extraction and qPCR

Colonic RNA was extracted utilizing TRIzol reagent and evaluated for purity as previously described [10]. The cDNA was prepared using iScript™ kit (Bio-Rad, Hercules, CA, USA), per the manufacturer’s instructions. Quantitative reverse transcription polymerase chain reaction (qRT-PCR) was conducted on a CFX384 real-time thermocycler (Bio-Rad, Hercules, CA, USA) with 18S rRNA serving as the housekeeping gene. Primers for PCR analysis of mRNA expression are listed in Supplementary Table S1. 

### 2.5. Immunoblotting Analysis

Western blot procedure was completed per established method [10]. In brief, lyophilized colonic tissue was ground into a fine powder and homogenized in a Precellys homogenizer with the addition of lysis buffer. Separation took place on SDS-PAGE, followed by transfer onto a nitrocellulose membrane. Primary antibodies against MATH1 (Atoh1) were purchased from DSHB (Iowa City, IA, USA). Primary antibodies were diluted 1:1000. The binding of antibodies was detected using enzyme horseradish peroxidase-coupled anti-mouse immunoglobulin and visualized using chemiluminescence. The density of bands was quantified and then normalized by referencing the β-tubulin content.

### 2.6. Metabolite Extraction and Derivatization

Cecal metabolites were extracted and processed according to a previously established method with minor modifications [27]. In brief, 50 mg of ground cecal content samples were extracted in 0.5 mL of methanol/H_2_O (8/2, *v*/*v*) containing 0.6 μg/mL ribitol. Samples were sonicated three times for 20 s. The samples were then centrifuged at 13,500 rpm for 15 min, and the resulting supernatant was dried under N_2_ gas.

Derivatization was first entailed by the addition of 25 μL freshly prepared methoxyamine hydrochloride (20 mg/mL) in pyridine, vortexed, and incubated at 60 °C for 60 min. This was followed by the addition of 25 μL MSTFA-1% TMCS, vortexing, and incubation at 60 °C for 30 min.

### 2.7. Metabolomics by GC-MS

Analysis of cecal metabolite profile was conducted on an Agilent 7890B GC system, coupled to an Agilent 5977A GC-MSD equipped with a HP-5 ms column (30 m × 250 μm i.d., 0.25 μm film thickness, Agilent Technologies, Palo Alto, CA, USA). Splitless injection, helium flow rate of 1.0 mL/min and the following temperature control protocol was used: hold at 50 °C for 2 min, increase 5 °C/min to 270 °C, increase 2.5 °C/min to 290 °C, increase 10 °C/min to 310 °C and hold 4 min. 

The data sets were first processed for peak deconvolution, compound identification and blank subtraction using the Unknowns Analysis tool of the MassHunter Quantitative Analysis software package (B.07, Agilent Technologies, Santa Clara, CA, USA). Compound identification was performed by searching the NIST11 mass spectral library. The identifications with scores of more than 70 were used for subsequent analyses. Alignment, normalization and statistical analysis were performed by Mass Profiler Professional Software (MPP) (12.6, Agilent Technologies, Santa Clara, CA, USA), a multivariate statistical analysis package, to find compounds present at different levels between treatments. Alignment was based on the compound name and retention time, and quantitation normalization was based on the peak ratio to the internal standard. Metaboanalyst was utilized for pathway analysis [27].

### 2.8. Statistical Analysis

Each individual mouse was considered as an experimental unit. Data were analyzed using General Linear Model [28]. All data were presented as mean ± standard error of mean (SEM). Treatment groups were analyzed utilizing 2-tailed Student’s *t* test. A significant difference was considered as *p* ≤ 0.05.

## 3. Results

### 3.1. Metabolome

**Figure 1:**
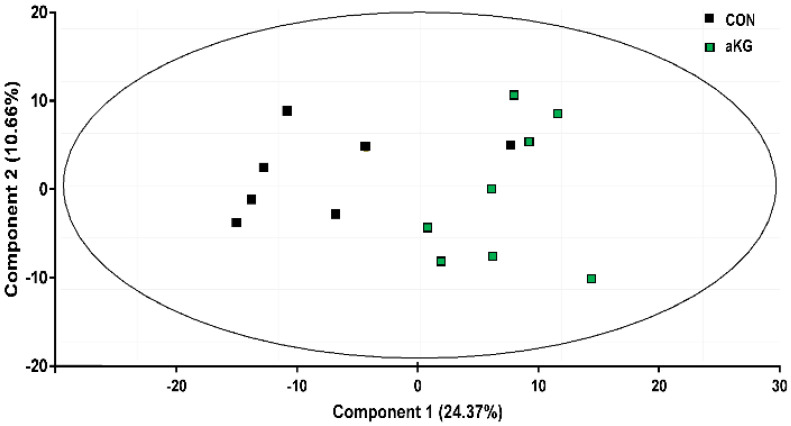
PCA of the cecal metabolome of dextran sulfate sodium (DSS)-induced colitis mice receiving water with or without alpha-ketoglutarate (aKG). CON: control mice without aKG supplementation in drinking water; aKG: mice receiving 1% aKG. *n* = 7–8 mice per group.

By utilizing GC-MS metabolomics, we analyzed the metabolites present in cecal content. Principal components analysis (PCA) revealed a separation between the aKG and CON treatment groups (Figure 1).

**Figure 2:**
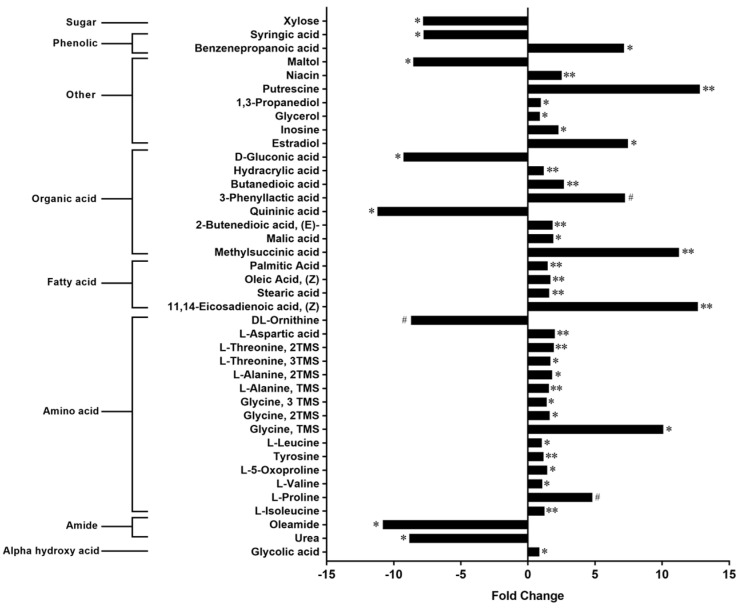
Cecal metabolite profiles in dextran sulfate sodium (DSS)-induced colitis mice receiving water with or without alpha-ketoglutarate. Fold changes related to CON, #: *p* < 0.10, *: *p* < 0.05, **: *p* < 0.01.

In total, the contents of 40 compounds were found to be different between the CON and aKG groups (Figure 2). These metabolites included amino acids, fatty acids, and the B vitamin, niacin. Dietary aKG-elevated cecal levels of succinic acid (butanedioic acid), the conjugate acid of succinate, by 2.7-fold (Figure 2). Putrescine, a polyamine produced by intestinal microbiota and methysuccinic acid were among the metabolites with the largest fold increases by dietary aKG: nearly 13-fold (Figure 2). Various amino acids, including essential amino acids and those involved in the TCA cycle, such as threonine and aspartic acid, were elevated in the aKG treatment group (Figure 2). The amino acid that showed the largest magnitude of expansion in the aKG treatment group was glycine, displaying an approximate 10-fold change (Figure 2). On the other hand, ornithine, an amino acid intermediate of the urea cycle [29], displayed a tendency of reduction in the aKG treatment group (Figure 2). Amide products such as oleamide and urea were significantly decreased in aKG supplemented group: 10.8 and 8.8-fold, respectively (Figure 2).

**Figure 3:**
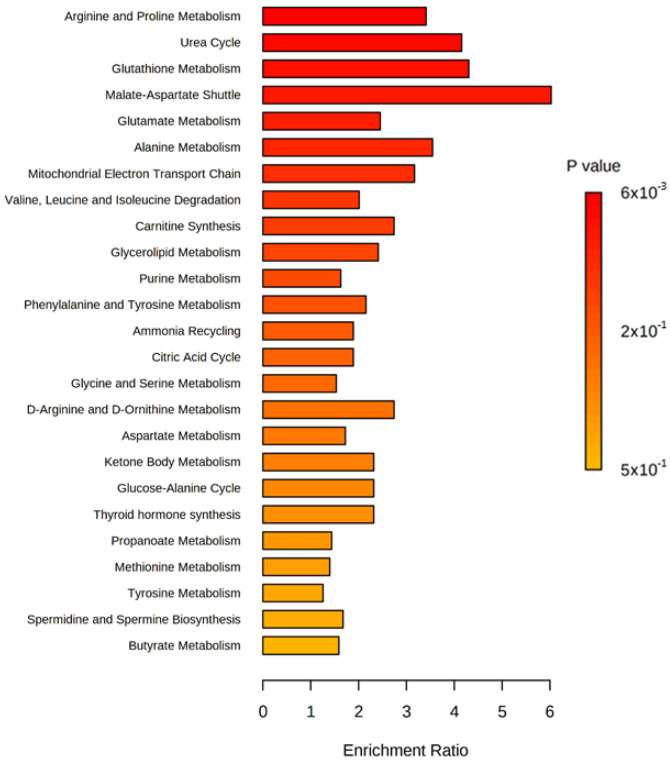
Pathway enrichment analysis of the differential metabolites in cecal samples of dextran sulfate sodium (DSS)-induced colitis mice receiving water with or without alpha-ketoglutarate.

Consistently, the pathway analysis of differential metabolites revealed that the effects of aKG supplementation were primarily associated with the malate-aspartate shuttle, the urea cycle, arginine and proline metabolism, and glutathione metabolism (Figure 3).

### 3.2. Goblet Cell Differentiation

**Figure 4:**
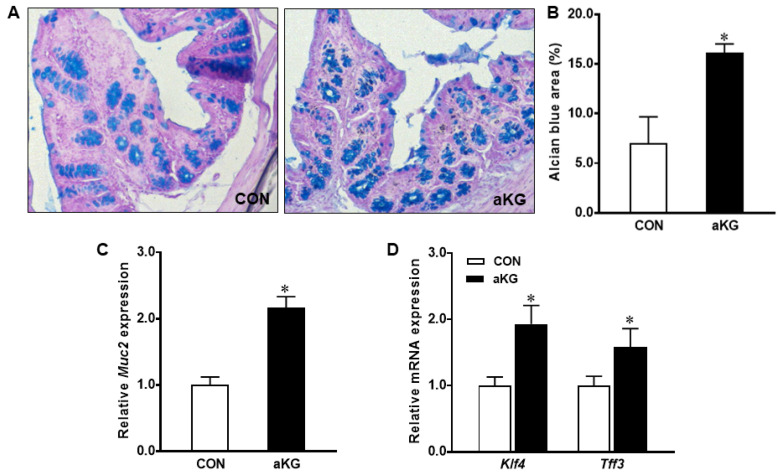
Goblet cells in dextran sulfate sodium (DSS)-induced colitis mice supplemented with or without alpha-ketoglutarate (aKG). (**A**) Representative Alcian blue staining; (**B**) Goblet cell density; (**C**) mRNA expression of *Muc2*; (**D**) mRNA expression of goblet cell differentiation markers. CON: control mice without aKG; aKG: mice receiving 1% aKG; Mean ± SEM, *n* = 4–7 mice per group. *: *p* < 0.05.

**Figure 5:**
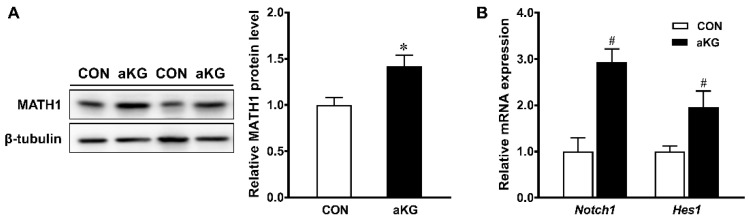
Alpha-ketoglutarate (aKG) supplementation upregulates signaling transcription factors committing cells to the secretory cell lineage. (**A**) MATH1 immunoblotting; (**B**) mRNA expression of *Notch1* and *Hes1*. CON: control mice without aKG; aKG: mice receiving 1% aKG in drinking water. Mean ± SEM, *n* = 7–8 mice per group. #: *p* < 0.10, *: *p* < 0.05.

The Alcian blue staining of colonic samples showed that aKG treatment promoted the differentiation of mucin-producing goblet cells (*p* < 0.05) (Figure 4A,B), associated with a markedly increased mRNA expression of *Muc2* (*p* < 0.01) (Figure 4C). Consistent with the increased amount of goblet cells, the aKG group had a greater expression of *Klf4* and *Tff3*, markers for goblet cell differentiation (*p* < 0.05) (Figure 4D). Protein levels of MATH1 were increased (*p* < 0.05) in the aKG group compared to the control, suggesting that aKG treatment promoted the differentiation of epithelial stem cells into the secretory lineage (Figure 5A). The mRNA level of *Hes1* and *Notch1* tended to be increased (*p* < 0.10), suggesting that the effects of aKG additionally promoted enterocyte differentiation and regenerative signaling (Figure 5B).

## 4. Discussion

The homeostasis of intestinal epithelium requires a careful balance of proliferation and differentiation, with differentiating cells undergoing a transition from glycolysis to oxidative phosphorylation [12]. Disturbances, such as hyperproliferation, increased susceptibility to carcinogenesis [30,31]. We reported that aKG ameliorates symptoms of DSS-induced colitis in mice by suppressing inflammation and promoting oxidative phosphorylation [10]. In the present study, we explored whether the increase in oxidative phosphorylation caused by aKG was paralleled by a change in the intestinal metabolome or an increase in differentiation signaling.

The intestine is a dynamic environment in which epithelial cells interact with immune cells and microorganisms [32], with microbial metabolites serving as mediators [33]. Compounds found in the cecum, reflect both host and microbial metabolism [34]. Consistent changes in metabolome were observed between cecal and fecal contents [35]. Certain microbial metabolites display harmful attributes, promoting mitochondrial dysfunction [36]. Thus, the microbiome plays an active role in directing energetics in the gut. The relationship works in both ways, as evidence points toward the anaerobic status of the gut lumen in shaping the microbiome [37].

The metabolome is known to be altered in experimental colitis. The TCA cycle metabolites, such as citric acid, fumaric acid, and succinic acid, are decreased in the serum of DSS-induced colitis [38]. Succinate can be produced from aKG by scavenging H_2_O_2_ [39], and mice provided aKG in drinking water possessed higher levels of succinate than the control group. Many amino acids also experience changes, either positive or negative, in the serum as well as colon tissue [38]. In patients with ulcerative colitis, lower levels of glycine and other amino acids are present in lesioned tissue compared to normal tissue [40]. Glycine plays a role in multiple biological functions, and its reduction is associated with metabolic disorders [41,42]. Glycine demonstrates a capacity to ameliorate experimentally induced colitis in rats [43]. Consistent with ameliorated colitis in aKG supplemented group [10] and the beneficial role of glycine against colitis, dietary aKG elevated cecal levels of glycine in DSS-induced colitis. Glycine plays an important role in glutathione synthesis [44,45]. In agreement with dramatically increased glycine, the pathway analysis of differential metabolites identified them to be enriched in glutathione metabolism. In support of our findings, glutathione was shown to be depleted in experimental colitis, and its restoration ameliorated mucosal damage [46].

In addition, threonine levels were markedly increased in colitis mice receiving aKG supplementation. In agreement with our findings, threonine supplementation mitigates colitis symptoms in DSS-induced rats [47]. The supplementation with amino acids such as threonine helps to ameliorate experimentally induced colitis through the restoration of the mucus layer on the epithelial surface [47]. Another beneficial metabolite that increased in the aKG group was niacin. Niacin plays a protective role against intestinal inflammation, acting as an agonist of the GPR109a receptor to suppress pro-inflammatory IL-23 [48].

Urea metabolism was also impacted by aKG supplementation, with the aKG group showing lower levels of urea and ornithine. Additional compounds related to urea metabolism include L-alanine, L-aspartic acid, and the TCA cycle intermediate, fumaric acid (2-Butenedioic acid (E)). The transamination of ammonia produces L-alanine, which, in the liver can react with aKG to produce glutamate and pyruvate [49]. Glycine, L-alanine, and L-5-oxoproline, which were elevated in the aKG treatment group, contributed to the enrichment of glutathione metabolism. Urea is known to impair epithelial barrier function through the downregulation of tight-junction proteins [50]. Increased levels of urea also suggest increased intestinal ammonia, which causes epithelial damage, depletes mucus, and promotes cell proliferation in the colon [51]. In agreement with our findings, previous work shows that the supplementation of aKG to growing pigs reduces levels of cecal ammonia compared to the controls [52]. As part of the urea cycle, ornithine serves as a precursor of polyamines, through the involvement of ornithine decarboxylase, which is elevated in tissues from patients with colitis [53]. Interestingly, putrescine, the initial polyamine formed from ornithine, by ornithine decarboxylase, was 10-fold elevated in the cecal content of the aKG treatment group. Putrescine can be absorbed from the colonic lumen by epithelial cells, activating oxidative phosphorylation, increasing anti-inflammatory M2 macrophage abundance, and lessening the severity of DSS-induced colitis symptoms [54]. The high level of putrescine detected in the aKG group provides a metabolomic explanation for the improved colitis symptoms, enhanced oxidative phosphorylation, and M1 to M2 macrophage polarization observed in aKG-treated colitis mice [10]. Increased putrescine and ornithine decarboxylase are associated with improved duodenal mucosal recovery in response to stress-induced damage [55]. In the small intestine, putrescine is converted into succinate and utilized as an energy source by enterocytes [56].

The supplementation with aKG also increased levels of inosine, succinate, and methylsuccinic acid. Inosine was reported to increase colonic differentiation through enhancing mitochondrial function and aerobic metabolism [57]. Urinary levels of this carboxylic acid were decreased in patients with irritable bowel syndrome compared to their healthy counterparts [58]. A recent study showed that succinate in the gut lumen prevents intestinal inflammation through the expansion of tuft cells [59]. These metabolomic findings provide additional insights into the beneficial effects of dietary aKG for ameliorating DSS-induced colitis symptoms [10].

Oxidative phosphorylation is vital for maintaining a functioning intestinal epithelium, which becomes progressively prominent in differentiating cells as they move further away from crypts [12]. The reduced mitochondrial ATP production impaired intestinal epithelial barrier function [60]. Given the important role of oxidative phosphorylation in dictating cell fates, we next explored the impact of dietary aKG on goblet cell differentiation. In colitis, IL-18 contributes to dysfunction by depleting goblet cells and weakening the mucosal barrier [61]. In agreement with decreased IL-18 and inflammation [10], dietary aKG increased the levels of colonic goblet cells in DSS-treated mice, indicating a stronger, protective mucus layer. The protein content of MATH1, which commits epithelial cells to the secretory lineage, was increased by dietary aKG. At the mRNA levels, we found that aKG supplementation increased levels of *Muc2*, *Tff3* and *Klf4*, transcription factors responsible for goblet cell differentiation. MUC2 is secretory mucin that exists in high levels in the colon under normal conditions, and is essential for maintaining intestinal homeostasis, exemplified by MUC2-deficient mice developing colitis [62]. Similarly, Tff3-deficient mice suffer worsened colitis symptoms following exposure to DSS, as well as the increased expansion of the proliferative compartment [63]. The transcription factor KLF4 is vital for the development of goblet cells and ensuring proper mucin production [18]. Klf4-deficient mice display normal levels of proliferation and the presence of most secretory intestinal cell types, but goblet cells are nearly absent [18]. The expression of *Klf4* is negatively regulated by Notch signaling, which encourages proliferation [19]. That being said, the antagonistic relationship between HES1 and MATH1 is primarily established in the small intestine not the colon, as *Hes1* null mice exhibit increased levels of MATH1 in the small intestine but not the large [64,65]. Dietary aKG supplementation tended to increase the mRNA levels of *Notch1* and its downstream target *Hes1*. Consistently, along with ameliorated symptoms and histological damage, mice subjected to DSS-induced colitis receiving 5% red raspberry displayed a greater goblet cell density and increased mRNA levels of *Muc2* and *Klf4*, and HES1 protein content [66]. The promotion of not only goblet cell differentiation, but also enterocyte differentiation may aid in the recovery of colonic inflammation. Along with changes to the metabolome, aKG supplementation fortified the intestinal environment by promoting differentiation.

## 5. Conclusions

Dietary aKG strengthens the epithelial barrier through the upregulation of transcription factors stimulating secretory cell differentiation, increasing goblet cell density and mucin production. GC-MS metabolomics revealed that the contents of 40 metabolites were altered in aKG supplemented groups. Most noticeably, putrescine, a compound recently reported to ameliorate colitis through the promotion of oxidative phosphorylation and M2 macrophage levels, was profoundly increased due to aKG supplementation. In addition, dietary aKG decreased the level of urea, a harmful compound known to impair the intestinal barrier. These provide metabolome-based explanations to the beneficial effects of aKG against experimental colitis.

## Data Availability

Not applicable.

## Supplementary Materials

The following supporting information can be downloaded at: https://www.mdpi.com/article/10.3390/nu14061148/s1, Table S1: Primer sequences used for qRT-PCR analyses [67,68].
