# Supplementary material for: Alpha-Ketoglutarate Promotes Goblet Cell Differentiation and Alters Urea Cycle Metabolites in DSS-Induced Colitis Mice
Source: Nutrients. 2022 Mar 9;14(6):1148. doi: 10.3390/nu14061148 (PMC8951758; doi:10.3390/nu14061148)
Supplement: Supplementary file 1 [file nutrients-14-01148-s001.zip › nutrients-1583893-supplementary.pdf]

Supplementary Materials

Table S1. Primer sequences used for qRT-PCR analyses.

Gene name	Accession No.	Product size	Sequence (5'→3')	Source
Hes1	NM_008235.2	86 bp	F: CAACACGACACCGGACAAAC<br>R: TTCTTGCCCTTCGCCTCTTC	[1]
Klf4	NM_010637.3	75 bp	F: CAGGATTCCATCCCCATCCG<br>R: GAGAGGGACTTGTGACTGC	[2]
Muc2	NM_023566.2	101 bp	F: ATGCCCACCTCCTCAAAGAC<br>R: GTAGTTTCCGTTGGAACAGTGAA	[2]
Notch1	NM_008714.3	268 bp	F: GCTCCGAGGAGATCAACGAG<br>R: TTGACATCACCCTCACACCG	This study
Tff3	NM_011575.2	103 bp	F: CTGTCACATCGGAGCCAGTGT<br>R: AATGTGCATTCTGTCTCCTGC	[2]
18S RNA	NM_003278.1	122 bp	F: AAGACGGACCAGAGCGAAAG<br>R: ATCGCCAGTCGGCATCGTTT	[2]

References

1. Sun, X.; Du, M.; Navarre, D.A.; Zhu, M.-J. Purple potato extract promotes intestinal epithelial differentiation and barrier function by activating AMP-activated protein kinase. *Mol Nutr Food Res* **2018**, *62*, 1700536, doi:10.1002/mnfr.201700536.
2. Bibi, S.; de Sousa Moraes, L.F.; Lebow, N.; Zhu, M.J. Dietary green pea protects against DSS-induced colitis in mice challenged with high-fat diet. *Nutrients* **2017**, *9*, 509, doi:10.3390/nu9050509.
